# When the pandemic opts for the lockdown: Secretion system evolution in the cholera bacterium

**DOI:** 10.15698/mic2021.03.744

**Published:** 2021-02-18

**Authors:** Francis J. Santoriello, Stefan Pukatzki

**Affiliations:** 1 Department of Immunology and Microbiology, University of Colorado Denver Anschutz Medical Campus, 13001 E 17th Pl, Aurora, CO 80045.; 2 Department of Biology, The City College of New York, 160 Convent Ave, New York, NY 10031.

**Keywords:** Vibrio cholerae, Type VI Secretion System, site-specific recombination, mobile DNA, prophage grounding, prophage domestication

## Abstract

*Vibrio cholerae*, the causative agent of the diarrheal disease cholera, is a microbe capable of inhabiting two different ecosystems: chitinous surfaces in brackish, estuarine waters and the epithelial lining of the human gastrointestinal tract. *V. cholerae* defends against competitive microorganisms with a contact-dependent, contractile killing machine called the type VI secretion system (T6SS) in each of these niches. The T6SS resembles an inverted T4 bacteriophage tail and is used to deliver toxic effector proteins into neighboring cells. Pandemic strains of *V. cholerae* encode a unique set of T6SS effector proteins, which may play a role in pathogenesis or pandemic spread. In our recent study (Santoriello *et al.* (2020), Nat Commun, doi: 10.1038/s41467-020-20012-7), using genomic and molecular biology tools, we demonstrated that the T6SS island Auxiliary Cluster 3 (Aux3) is unique to pandemic strains of *V. cholerae*. We went on to show that Aux3 is related to a phage-like element circulating in environmental *V. cholerae* strains and that two genetic domestication events formed the pandemic Aux3 cluster during the evolution of the pandemic clone. Our findings support two main conclusions: (1) Aux3 evolution from phage-like element to T6SS cluster offers a snapshot of phage domestication in early T6SS evolution and (2) chromosomal maintenance of Aux3 was advantageous to the common ancestor of *V. cholerae* pandemic strains.

While pandemic pathogens often appear suddenly and move swiftly throughout the global population, they are not unpredictable. Most infectious agents with pandemic potential first circulate in an environmental reservoir before spilling over into the human population. Studying the evolutionary steps at play in the early development of a pandemic is vital for understanding how pathogens make the jump between reservoir and host and indicates factors that can be targeted for pandemic prevention or mitigation. Descendants of a single ancestral O1 *V. cholerae* clone have caused the seven recorded pandemics since the early 1800s. Classical biotype strains caused the first through sixth pandemics, and El Tor biotype strains cause the ongoing seventh pandemic. Pandemic strains of *V. cholerae* cause cholera's characteristic secretory diarrhea with the virulence factors cholera toxin (CT) and toxin co-regulated pilus (TCP). These factors alone, however, do not make a strain pandemic; several non-O1 strains of *V. cholerae* and the close relative *Vibrio mimicus* encode CT and TCP and only cause isolated disease. The full set of factors necessary for the pandemic spread of *V. cholerae* remains unknown. In Santoriello *et al.,* 2020, we indicate T6SS cluster Aux3 in pandemic *V. cholerae* as a potential member of this list.

All *V. cholerae* strains for which genomic sequences are available carry a contact-dependent T6SS encoded over three core genetic loci: a large cluster and two auxiliary clusters (Aux1 and Aux2). Most T6SS cluster genes are homologous to bacteriophage structural genes, indicating that the T6SS is the degraded remnant of a prophage infection. While these clusters are phage-related, they lack phage regulators and recombination machinery. Each gene cluster also encodes a distinct effector/immunity module. Thus, each *V. cholerae* strain delivers a set of three T6SS effectors with this killing machine. Cells with complementary effector sets are protected from cross-killing by the cognate immunity proteins, but a different effector/immunity type at any of the three clusters will lead to competition between strains. Previous work from our lab and Yann Boucher's group at the University of Alberta shows that effector sets in the environmental *V. cholerae* population are highly variable from strain to strain. All pandemic strains, however, encode an identical, unique set of effectors. This phenomenon led us to question whether specific T6SS effectors were necessary to develop pandemic *V. cholerae*.

In 2015, the Mekalanos group at Harvard Medical School identified a third auxiliary T6SS cluster (Aux3) in O1 *V. cholerae* strains 2740-80 and V52, close relatives of the O1 El Tor and O1 Classical *V. cholerae* clades, respectively. In Santoriello *et al.*, 2020, we show that pandemic *V. cholerae* strains of both the El Tor and Classical lineages are significantly enriched (566 out of 572) for the Aux3 cluster, indicating that Aux3 has been maintained over all seven pandemics. Further, we show that the Aux3 cluster is mostly absent from environmental *V. cholerae* strains with the exception of nine isolates. The Aux3 cluster found in environmental isolates is unique. It encodes ~40 additional genes when compared to the Aux3 cluster of pandemic strains, the majority of which are homologs to bacteriophage structural and regulatory genes (**Fig. 1**). By comparing the presence or absence of Aux3 to a phylogenetic tree of pandemic *V. cholerae*, environmental *V. cholerae*, and closely related Vibrios, we show that the long form of Aux3 is sporadically dispersed in the environmental strains, indicating lateral transfer of Aux3 in the aquatic reservoir (**Fig. 1**). A common ancestor of the pandemic clade and its closely related environmental sister clade acquired the long, prophage-like Aux3, and this module was then degraded down to the short Aux3 in the common pandemic ancestor (**Fig. 1**). Based on these findings, we hypothesize that a prophage-like element circulating in the environmental reservoir gave rise to the Aux3 cluster – the only T6SS cluster that is mostly unique to pandemic *V. cholerae*. We believe that Aux3 evolution mirrors early T6SS evolution in which a prophage infection was degraded to only the components necessary to confer a fitness advantage.

**Figure 1:**
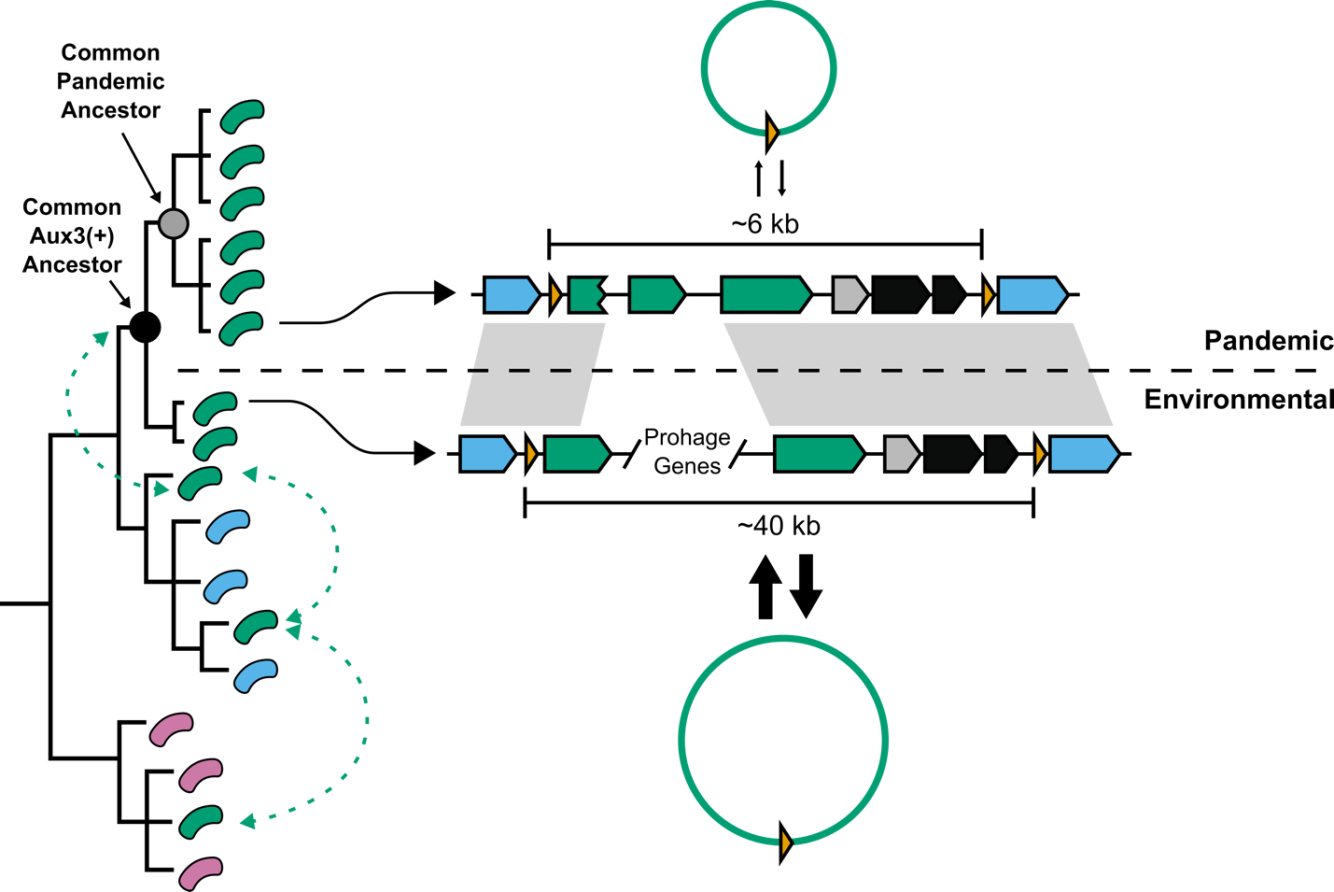
FIGURE 1: Schematic of T6SS cluster Aux3 domestication in the transition from the environmental to the pandemic population of *V. cholerae*. (Left) Theoretical phylogenetic tree of the *V. cholerae* population and other closely related Vibrios. Green cells = Aux3(+) isolates. Blue cells = Aux3(-) *V. cholerae*. Purple cells = close relative, Aux3(-) Vibrios. Green dashed arrows = theoretical Aux3 lateral transfer events. (Right) Schematic of environmental (bottom) and pandemic (top) Aux3 clusters. The weight of the arrows between the linear and circular Aux3 modules represent the excision quantity. Grey boxes represent homologous regions between pandemic Aux3 and environmental Aux3. Black dot represents the common ancestor of the pandemic clade and its closely-related environmental sister clade. Grey dot represents the common ancestor of the pandemic clade.

We further show that, unlike the other T6SS clusters in *V. cholerae*, both forms of the Aux3 cluster encode a site-specific recombinase and are flanked by repeat elements called attachment sites (*att*). Each form of Aux3 can be excised from the *V. cholerae* chromosome to form a circular DNA element, but environmental strains encoding the prophage-like form of Aux3 excise the cluster at significantly higher levels than pandemic strains encoding the short form. By aligning the environmental and pandemic Aux3 clusters against each other, we were able to identify two key mutations that resulted in lower excision levels in pandemic strains: (1) the C-terminal end of the Aux3 integrase is truncated in pandemic *V. cholerae* and (2) pandemic Aux3 lacks a recombination directionality factor (RDF) present in all environmental Aux3 clusters (**Fig. 1**). Truncation of the Aux3 integrase likely leads to reduced activity, and loss of an RDF will shift any remaining catalytic activity towards integration rather than excision. Importantly, expression of the full-length integrase and the RDF from environmental *V. cholerae* in a pandemic strain background is sufficient to induce environmental levels of excision. This dual mechanism of prophage domestication leading to a chromosomal lockdown of the Aux3 element in pandemic *V. cholerae* supports a selective pressure to maintain the Aux3 cluster on the *V. cholerae* chromosome.

Together, our data support selection for Aux3 domestication and maintenance during development of the pandemic clade of *V. cholerae*, but whether and how the Aux3 genes contribute to pandemic development remains unknown. In 2020, the Aux3 effector protein, TseH, was shown to have species-specific bacteriolytic activity by Tao Dong's group at the University of Calgary. *V. cholerae* with TseH as its only active T6SS effector rapidly kills Aeromonas and Edwardsiella isolates in a T6SS-dependent manner. Both Aeromonas and Edwardsiella species are commonly isolated from fresh and brackish water samples, indicating that *V. cholerae* may interact with these organisms in its environmental reservoir. It is possible that a competitive edge over environmental competitors of the Aeromonas and Edwardsiella genus simply led to a greater abundance of the pre-pandemic ancestor in the aquatic reservoir. It is important to consider, however, that free-swimming bacteria cannot kill with the T6SS. Instead, bacteria must be in contact with a surface to effectively kill competitors in a contact-dependent manner. It follows to investigate the specific surfaces or environmental niches co-colonized by these species because our genomic evidence supports the selection for factors like Aux3 that allow *V. cholerae* to be competitive in these spaces. Further, we believe it is crucial to determine the effects that colonization of these niches have on the physiology and transmission dynamics of *V. cholerae* bacteria. For instance, Aeromonas and Edwardsiella are common fish pathogens. TseH may confer a competitive edge to *V. cholerae* in the colonization of fish. Passage through vertebrate hosts (mice, rabbits, and humans) has been shown to induce *V. cholerae* aggregate formation and hyper-motility, two states that increase the likelihood of transmission between hosts. If TseH drives colonization of marine vertebrates such as fish through T6SS-dependent competition, it may also indirectly lead to increased transmission of Aux3(+) *V. cholerae* (**Fig. 2**). Adaptation to marine vertebrate hosts could also have led to the accidental, parallel adaptation to the human gastrointestinal tract. Our combined genomic and molecular genetics approach indicates Aux3 as an island of potential pandemic importance based on evolutionary patterns, enabling us to formulate these questions about physiological significance. While our studies do not fully implicate Aux3 and its effector TseH as a pandemic factor, they justify further mechanistic studies into its role in the various contexts of the *V. cholerae* life cycle.

**Figure 2:**
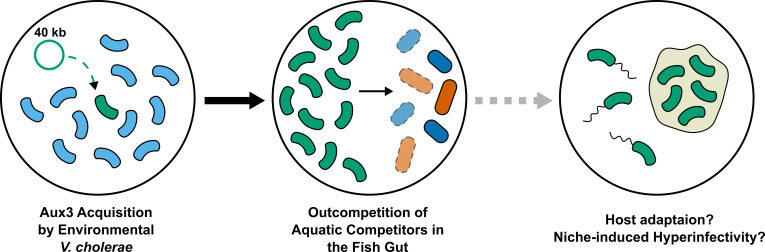
FIGURE 2: Known and hypothetical advantages of Aux3 acquisition by *V. cholerae*. (Left) Environmental *V. cholerae* laterally acquires Aux3. Light blue curved cells = Aux3(−) *V. cholerae*. Green curved cells = Aux3 (+) *V. cholerae*. (Middle) Aux3(+) *V. cholerae* kills Aeromonas (red cells) and Edwardsiella (dark blue cells). (Right) If Aux3-dependent killing is important for colonization a specific niche such as the fish gut, then passage through that niche may induce physiological changes important for pathogenesis such as hypermotility or aggregate formation.

